# Culture Shapes Efficiency of Facial Age Judgments

**DOI:** 10.1371/journal.pone.0011679

**Published:** 2010-07-21

**Authors:** Gizelle Anzures, Liezhong Ge, Zhe Wang, Shoji Itakura, Kang Lee

**Affiliations:** 1 Department of Human Development and Applied Psychology, University of Toronto, Toronto, Canada; 2 Department of Psychology, Zhejiang Sci-Tech University, Hangzhou, People's Republic of China; 3 Department of Psychology, Kyoto University, Kyoto, Japan; University of Leuven, Belgium

## Abstract

**Background:**

Cultural differences in socialization can lead to characteristic differences in how we perceive the world. Consistent with this influence of differential experience, our perception of faces (e.g., preference, recognition ability) is shaped by our previous experience with different groups of individuals.

**Methodology/Principal Findings:**

Here, we examined whether cultural differences in social practices influence our perception of faces. Japanese, Chinese, and Asian-Canadian young adults made relative age judgments (i.e., which of these two faces is older?) for East Asian faces. Cross-cultural differences in the emphasis on respect for older individuals was reflected in participants' latency in facial age judgments for middle-age adult faces—with the Japanese young adults performing the fastest, followed by the Chinese, then the Asian-Canadians. In addition, consistent with the differential behavioural and linguistic markers used in the Japanese culture when interacting with individuals younger than oneself, only the Japanese young adults showed an advantage in judging the relative age of children's faces.

**Conclusions/Significance:**

Our results show that different sociocultural practices shape our efficiency in processing facial age information. The impact of culture may potentially calibrate other aspects of face processing.

## Introduction

The ability to process faces is crucial in our daily social interactions; deficits in this ability lead to debilitating social consequences as in the case of autism or prosopagnosia. Given the importance of face processing in our social interactions, it is reasonable to assume that social interactions, in turn, influence how we process faces. Indeed, extensive research has established that differences in the quantity of social interactions fine tunes one's visual processing of faces. For example, an abundance of interaction with own-race individuals and limited experience with other-race individuals lead to better memory for own-race faces relative to other-race faces [1]. However, recent evidence of cross-cultural differences in face scanning has led to the speculation that cultural differences in social practices may also influence how we process faces [2]. In addition, the existing cross-cultural literature shows that differential experience at the level of both the physical and the sociocultural environment shapes our general visual perception of the world [3]–[9]. Here, we provide the first evidence that sociocultural practices also play a role in shaping our perception of faces.

From among the various types of information that one can abstract from a face, we specifically focus on facial age perception because of marked cultural differences in the amount of emphasis placed on the age of social partners. Thus, exposure to unique cultural practices resulting in differential emphasis on facial age might cultivate different levels of sophistication in processing age-related information. In the Japanese culture, an age hierarchy is well entrenched in everyday social interactions such that deference towards older individuals exists not only at the behavioural level – in the form of bowing and gaze aversion [10] – but also at the linguistic level. In Japan, one must use qualitatively different ways of speaking to individuals from different age groups [11]. Respect for an acquaintance even one year older than oneself requires the use of a polite form of speech. A more polite, honorific, form is used to show respect for even older individuals. Distinct sets of syntactic and semantic rules in the Japanese language specifically dictate how one should use these polite forms when interacting with older individuals. In contrast, a more casual way of speaking (e.g., use of slang, bluntness) is appropriate for close peers, and such speech is, in turn, slightly more casual and direct when speaking to younger individuals. In the Chinese culture, although respect for older individuals is also emphasized [12], such emphasis on respect appears to be of a relatively lesser degree than in Japan. Respectful speech in the Chinese culture is limited to one linguistic marker (i.e., a casual and polite form of the word “you”) that is reserved for senior citizens. In contrast, in North America, such respect is emphasized to an even lesser degree. There exist no established behavioural or linguistic displays of such respect, and disrespect towards the elderly is common [13].

These unique cultural differences in social interactions with different age groups may very well result in differences in processing facial age information. For example, the Japanese custom of differentially interacting with younger, older, and same-age individuals might cultivate more sophisticated processing of facial age information for younger, older, and same-age individuals relative to the Chinese and North American cultures. In contrast, relative to the North American culture, the Chinese culture which only places emphasis on respect for older individuals, might only cultivate more sophisticated processing of facial age information for adults rather than children.

To investigate the role of differential sociocultural experiences on facial age perception, Japanese, Chinese, and Asian-Canadian young adults were recruited for the present study. Adults are generally accurate in judging the age (i.e., in years) of an own-race individual by examining their face [14]–[17]. Thus, the present study examined whether differential sociocultural experiences influence fine-grained facial age perception by asking participants to make relative age judgments for male Asian faces. If facial age perception is influenced by differential culturally dictated emphasis on the age of social partners, then Japanese participants should be most proficient in their age judgments, followed by the Chinese participants, then the Asian-Canadians.

## Methods

### Ethics Statement

All procedures used in the current study were approved by the University of Toronto Research Ethics Board. Informed written consent was obtained from all participants involved in the study.

### Participants

Thirty-two Japanese adults (*M* = 22.84 years, *SD* = 2.62 years, 15 males) living in Japan, 39 Chinese adults (*M* = 21.54 years, *SD* = 1.10 years, 18 males) living in China, and 33 Asian-Canadians (*M* = 21.00 years, *SD* = 2.06 years, 3 males) participated in the study. Participants from all three sites were undergraduate students from Kyoto University, Zhejiang Sci-tech University, or the University of Toronto. Participants were given an honorarium for their participation.

Of the 33 Asian-Canadians, 22 were of Chinese descent, and the remaining were of Korean, Filipino, Japanese, Vietnamese, or mixed Asian descent. Three of the Asian-Canadian participants were international students, 10 were Asian-Canadian immigrants, and the remaining did not volunteer information regarding their status in Canada. The Asian-Canadian participants had been living in Canada for an average of nine years (*SD* = 4.65) and gave overall neutral ratings on a five-point scale to questions that inquired whether they often behaved in ways that are typical of their heritage culture (*M* = 3.31, *SD* = 1.00) and whether it was important for them to maintain or develop the practices of their heritage culture (*M* = 3.75, *SD* = 1.05). Thus, at least to some extent, the Asian-Canadians in the present sample appear to have acculturated to North American society.

### Stimuli

Twenty Asian male adult faces (i.e., 31- to 40-year-olds, *M* = 34.95, *SD* = 2.82) were used to create an averaged “100% Old” East Asian male adult face, and twenty Asian male children's faces (i.e., 11- to 12-year-olds, *M* = 11.5, *SD* = .51) were used to create an averaged “0% Old” East Asian male child face. All of the models that were used to create the stimuli were of Chinese descent. The averaged faces were created to control for individual differences in facial growth within a given age group. That is, our averaged male adult face is likely more representative of the male middle-age adult facial age group relative to the individual male adult faces that were used to create the averaged face. Our averaged male child face is likely also more representative of the male pre-adolescent facial age group relative to the individual faces that were used to create the averaged face.

**Figure 1:**
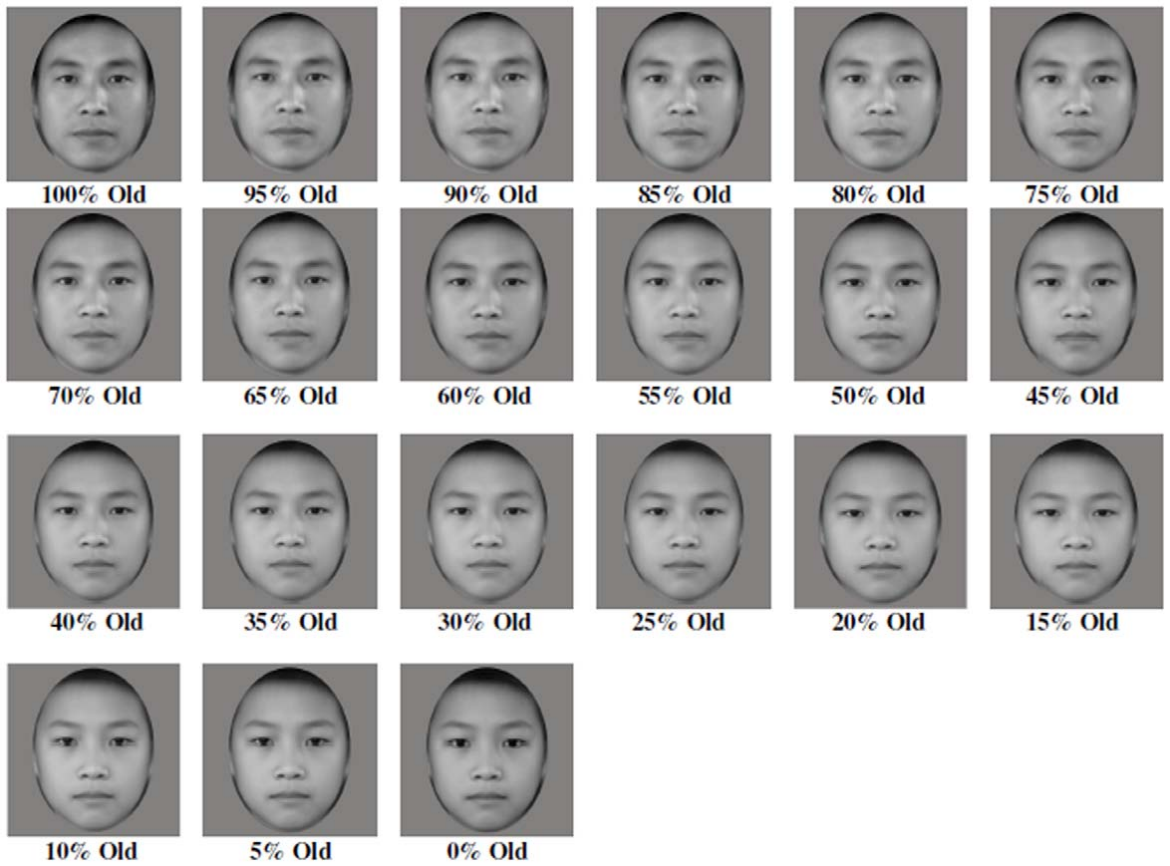
Stimulus set of Asian male faces.

The 100% Old (i.e., adult's face) and 0% Old (i.e., child's face) average faces were then averaged together in 5% increments to make additional composite faces (i.e., 21 composite faces in total) with varying degrees of old/young facial information that ranged from 100% Old to 0% Old (see Figure 1). All photos were presented in grayscale.

### Procedure

Participants were seated about 30 cm away from a 17-inch computer screen on which the stimuli (13.31° visual angle for the vertical dimension; 10.85° visual angle for the horizontal dimension) were presented. Participants were presented with randomly ordered trials that each showed two faces belonging to the same stimulus age group. Children face pairs included the 0%–30% Old faces, young adult face pairs included the 35%–65% Old faces, and middle-age adult face pairs included the 70%–100% Old faces. Within each stimulus age group, each face was paired with every other face four times (i.e., 84 trials per stimulus age group). Participants were asked to indicate which face in each pair was older via a key press. Each trial was preceded by a 500 ms crosshair, followed by a face pair presented for a maximum of 10 seconds or until a response was made. Prior to the age judgment task, Asian-Canadians were also asked to complete a questionnaire that inquired about their ethnic backgrounds and gauged their degree of acculturation into Western society.

The procedure described above was part of a larger study that involved additional tasks pertaining to adults' facial age judgment ability. In addition to making relative age judgments for stimulus faces belonging to the same age group, in 4 control trials, participants also made relative age judgments for the 100% Old average male adult face paired with the 0% Old average male child face. These 100% Old versus 0% Old face trials were interspersed across the child, young adult, and middle-age adult face trials.

## Results

Analyses of participants' accuracy and reaction time in relative facial age judgments for children (0%–30% Old), young adults (35%–65% Old), and middle-age adults (70%–100% Old) show that Japanese and Chinese participants were faster but less accurate than the Asian-Canadians (see Table 1). However, Japanese, Chinese, and Asian-Canadian participants showed a significant speed-accuracy tradeoff in their age judgments (*r* = .50, *p*<05, *r* = .53, *p*<05, and *r* = .49, *p*<05 respectively). Thus, inverse efficiency scores [18] expressed in ms were computed to account for the speed-accuracy tradeoffs – that is, reaction time scores for each stimulus facial age group were divided by their corresponding proportion correct score so that differences in reaction time performance decrease if differences in accuracy are large but remain the same if accuracy is identical. These inverse efficiency scores were then used in the subsequent analyses.

**Table 1:** Participants' accuracy and reaction time scores in milliseconds for child, young adult, middle-age adult, and control facial age judgment trials (i.e., prior to adjustment for speed-accuracy tradeoffs).

		Accuracy		Reaction Time	
Stimulus Facial Age Group	Participant Ethnicity	Mean	SD	Mean	SD
**Experimental Trials**					
**Child**	Japanese	.54	.06	1452.98	632.13
	Chinese	.49	.08	1866.74	761.35
	Asian-Canadians	.62	.09	2520.70	798.20
**Young Adult**	Japanese	.79	.06	1157.18	481.16
	Chinese	.79	.07	1426.19	505.31
	Asian-Canadians	.83	.05	1807.48	583.81
**Middle-age Adult**	Japanese	.81	.06	1100.34	404.84
	Chinese	.78	.05	1317.10	460.44
	Asian-Canadians	.85	.05	1723.38	584.37
**Control Trials**					
	Japanese	.98	.06	652.88	165.83
	Chinese	.99	.08	762.97	231.84
	Asian-Canadians	.99	.04	798.02	354.76

To determine whether differential sociocultural experience influences relative facial age judgments, an ANOVA was conducted with stimulus facial age (i.e., children, young adult, and middle-age adult) as a within-subjects factor, participant ethnicity as a between-subjects factor, and adjusted reaction time (i.e., inverse efficiency scores) as the dependent variable. The results revealed significant main effects of facial age, *F* (1, 112) = 263.49, *p*<.001, partial η^2^ = .72, and participant ethnicity, *F* (2, 101) = 11.09, *p*<.001, partial η^2^ = .18. Overall, participants were significantly faster in their relative age judgments for young adult faces than for children's faces *F*(1, 101) = 272.04, *p*<.001, and faster for middle-age adult than for young adult faces *F*(1, 101) = 13.73, *p*<.001. Japanese participants were also significantly faster than the Chinese and the Asian-Canadians in their relative age judgments (*p* values<.05).

**Figure 2:**
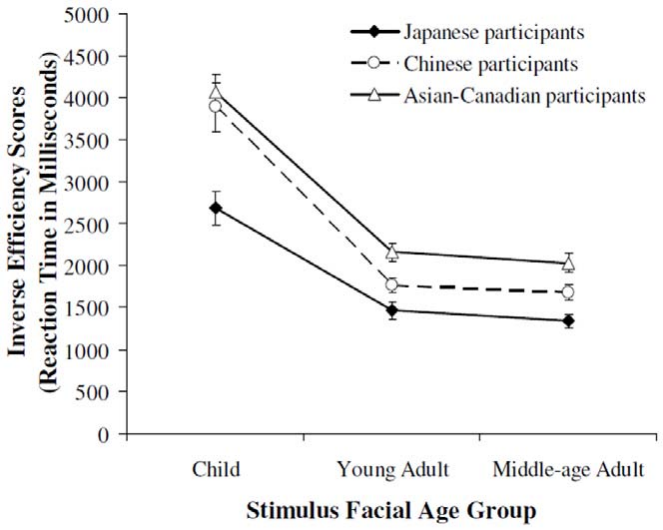
Participants' adjusted reaction time (i.e., inverse efficiency scores) in age judgments for child, young adult, and middle-age adult faces.

However, there was a significant interaction between stimulus facial age and participant ethnicity, *F* (2, 112) = 5.63, *p*<.05, partial η^2^ = .10 (see Figure 2). Japanese participants were significantly faster than the Chinese and the Asian-Canadians in their age judgments for children's faces, *t*(69) = 3.26, *p*<.05, and *t*(63) = 4.61, *p*<.001 respectively. Japanese and Chinese participants were comparable in the speed of their age judgments for young adult faces (*p*>.05), but both groups were significantly faster than the Asian-Canadians, *t*(63) = 4.61, *p*<.001, and *t*(70) = 2.84, *p*<.05. In addition, Japanese participants were fastest in their age judgments for middle-age faces, *t*(69) = 2.81, *p*<.05 and *t*(63) = 4.88, *p*<.001 compared to Chinese and Asian-Canadians respectively, followed by the Chinese, *t*(70) = 2.38, *p*<.05, then the Asian-Canadians.

It is also of interest to note that there was a statistically significant relationship between the number of years that the young adult Asian-Canadian participants had lived in North America and their efficiency in judging the facial age of relatively older middle-age adult faces. The longer the Asian-Canadian participants had lived in North America, the longer their response times in making facial age judgments for middle-age adult faces (*r* = .33, *p* = .05). Thus, it appears that as participants become more acculturated to North American society which does not emphasize respect for older individuals, the less efficient they are at processing facial age information for individuals older than themselves.

With regards to the control trials of the 100% Old middle-age adult face versus the 0% Old child face, participants in the three groups were highly accurate as expected. An ANOVA showed no difference in accuracy among the three groups (*p*>.05). An ANOVA on the raw reaction time data in the control trials revealed no significant difference in performance between the Japanese (*M* = 652.88 ms, *SD* = 165.83 ms), Chinese (*M* = 762.97 ms, *SD* = 231.84 ms), and Asian-Canadian (*M* = 798.02 ms, *SD* = 354.76 ms) participants (*p*>.05, see Table 1). A separate ANOVA using inverse efficiency scores for the control trials also revealed no significant difference in performance between the Japanese (*M* = 664.98 ms, *SD* = 170.12 ms), Chinese (*M* = 805.74 ms, *SD* = 452.33 ms), and Asian-Canadian (*M* = 805.00 ms, *SD* = 354.86 ms) participants (*p*>.05).

Thus, the group differences in the speed with which participants made their facial age judgments appear to be limited to small differences in facial age. Discrimination based on extreme facial age differences was comparable across the three ethnic groups, and appeared not to have been influenced by differential sociocultural experiences. Comparable performance for face pair trials with large facial age differences also suggests that the differences across the Japanese, Chinese, and Asian-Canadian participants for trials with faces from the same stimulus facial age group were not due to group differences in motivation or general reaction time.

## Discussion

Overall, the results of the present study show that differential sociocultural experiences do, indeed, have an influence on our visual processing of facial age. Japanese participants who experience greater sociocultural need to identify the age of their social partners were overall faster in their facial age judgments compared to the Chinese and Asian-Canadian participants. Thus, greater sociocultural emphasis in considering the age of social partners leads to an increased efficiency in processing facial age information. Although individuals most likely also rely on feedback regarding the age of their social partners (e.g., via age-related information exchanged during such interactions), the present study suggests that culturally distinct experiences also influences one's processing of facial age information.

Moreover, an examination of the interaction between participant ethnicity and stimulus facial age shows a more refined influence of differential sociocultural experience on facial age judgments. Japanese participants were fastest in their age judgments for children's faces, likely due to their socially constrained need to also consider the age of social partners younger than themselves. Thus, although the Japanese culture places a great degree of emphasis on respect towards individuals older than oneself, it appears that the linguistic manners used in interactions with individuals younger than oneself (e.g., use of slang and bluntness) may also enhance young adults' efficiency of age judgments for children's faces. In contrast, Chinese and Asian-Canadian participants who use no behavioural or linguistic markers when interacting with individuals younger than themselves showed no such advantage in their age judgments for children's faces. In addition, age judgments for young adult faces were more comparable across ethnic groups, likely due to the young adult participants' extensive experience with own-age peers [19]–[21]. However, Japanese and Chinese participants whose cultures attach greater importance to the age of their social partners were still significantly faster than the Asian-Canadians in their age judgments for own-age young adult faces.

Perhaps the most clear-cut example of the influence of differential sociocultural experience on facial age processing is evident in young adults' age judgments for older middle-age stimulus faces. Japanese participants whose culture places the greatest emphasis on respect for older individuals were faster than the Chinese and the Asian-Canadian participants. Chinese participants whose culture emphasizes respect for older individuals to a greater degree than North American culture (but to a lesser degree than the Japanese) were significantly faster than the Asian-Canadians in their age judgments for middle-age faces. Interestingly, Asian-Canadian young adult participants who had lived in North America for a longer time also tended to show slower response times in their age judgments for the middle-age adult faces. This tendency might be related to North American society's relative lack of emphasis on respect for older individuals. Thus, acculturation to North American society might be associated with less efficient processing of facial age information for individuals older than oneself.

A potential alternative explanation to our findings is group-related differences in processing speed across our Japanese, Chinese, and North American samples. More specifically, the main effect of participant ethnicity may be due to generally faster processing speed of Japanese participants. However, the interaction between participant ethnicity and stimulus facial age shows that although the Japanese were fastest in their age judgments for children's faces and middle-age adult faces, they were comparable to the Chinese participants in their age judgments of young adult faces. Performance on the control age judgment trials that compared the youngest average face with the oldest average face also showed no difference across the Japanese, Chinese, and Asian-Canadian participants. Thus, it is unlikely that the group differences in performance were due to general differences in processing speed. Such differences in performance are, instead, likely shaped by cultural differences in social interactions.

Overall, our results provide the first evidence of a link between social practices and face processing. This finding suggests that cultural practices play an important role in our perception of socially significant stimuli in our environment. Such practices perhaps calibrate our visual system to attend to, and develop expertise for, the culturally significant aspects of social stimuli. More broadly, our findings combined with findings from cultural psychological research suggest that cultural practices calibrate the manner in which we not only see the world [3]–[8], but also how we reason [22] and explain behaviour [23]. This culture-specific calibration likely leads to the development of optimal interactions with the social partners of one's own culture.
